# Protecting public health in mining: a Q-methodology examination of policy space and reform pathways in Tanzania

**DOI:** 10.1186/s12889-026-28398-7

**Published:** 2026-07-27

**Authors:** Joschka J. Proksik, Fritz Brugger, Gregory Kabadi, Isaac Lyatuu

**Affiliations:** 1 Department of Humanities, Social and Pol. Sc., Development Economics Group, ETH Zurich, Zurich, 8092 Switzerland; 2 Ifakara Health Institute, Dar Es Salaam, Tanzania

**Keywords:** Public health, Industrial mining, Tanzania, Health Impact Assessment, Environmental Impact Assessment, Q-methodology, Corporate Responsibility

## Abstract

**Background:**

Mining is a driver of economic development in low and lower-middle-income countries, particularly in sub-Saharan Africa. In Tanzania, the mining sector’s contribution to GDP is rising rapidly; digitalisation and the energy transition are driving demand. This growth entails significant risks to the environment, social well-being, and public health. Despite mandatory Environmental Impact Assessments, environmental outcomes remain weak and public health receives limited attention. Drawing on the expertise and experience of stakeholders involved in implementing impact assessment in Tanzania, this study identifies measures to improve outcomes.

**Methods:**

Q-methodology is used to elicit stakeholder perspectives on the most effective measures to advance public health protection within Tanzania’s industrial mining sector. Stakeholders were selected across different sectors to reflect a wide range of expertise, experience and viewpoints. Data were analysed to identify areas of consensus and divergence among stakeholders.

**Results:**

The study finds a shared recognition of the need for reform to strengthen public health regulation. Yet, study participants do not see merit in broad legal reforms such as introducing mandatory health impact assessments or overhauling environmental impact assessment legislation. The emphasis is on making the existing rules work as intended. Three perspectives dominate: The *Compliance Perspective* prioritises stronger public monitoring, enforcement, and sanctions regimes, with mining companies bearing the costs. The *Local Perspective* emphasises the need to shift resources and competences for monitoring to subnational authorities and to strengthen accountability towards communities. The *Partnership Perspective,* representing the smallest number of study participants, favours operational guidance documents on addressing public health in impact assessments and the active involvement of companies in the delivery of health services.

**Conclusions:**

Stakeholders identify a clear policy space for reform to strengthen public health protection in Tanzania’s mining sector and favour measures that improve the implementation of existing regulations. Priority actions include baseline health data collection, stronger consultation and grievance mechanisms, transparent monitoring and reporting, improved inter-agency coordination, and closer collaboration between mining companies and local health services. These findings suggest that implementation-focused reforms are more politically feasible and promise better outcomes than comprehensive regulatory change.

**Supplementary Information:**

The online version contains supplementary material available at https://doi.org/10.1186/s12889-026-28398-7.

## Background

Mining is a major source of revenue for many low and lower-middle-income countries, particularly in sub-Saharan Africa. The transition toward cleaner energy sources, combined with accelerated digitalisation, is expected to spur demand for critical minerals and further increase the significance of mining [22]. Tanzania is a case in point. The country holds substantial critical minerals, including graphite, nickel, lithium and rare earths, and the government is determined to capitalise on rising demand. In 2020, 70% of exploration licenses were granted for critical minerals [53]. The 2021 to 2025 development plan aimed at raising the mining sector’s GDP contribution from 5.6% in 2020 to 10% by 2025 and extractive foreign exchange earnings from US$3bn to US$5.5bn ([38] p. 94).

While mining revenues can support the Sustainable Development Goals, mining also poses significant threats to sustainability, including environmental damage, social disruption and harm to public health. The literature documents adverse impacts on water, soil and air quality, and on resource use in operational areas [44, 61].

To mitigate environmental harms, most countries have enacted legislation requiring environmental impact assessments (EIAs) for large-scale industrial mining projects [52, 59]. EIAs must be carried out by registered experts and require public consultation during scoping. Most countries, including Tanzania, Mozambique, Zambia, and South Africa, among others, have procedures comparable to those in industrialised countries such as the UK or the US [35, 41, 51]. Public health impacts, by contrast, often remain disregarded in legislation [66, 67]. Similar to EIA, Health Impact Assessment (HIA) is widely regarded as the most comprehensive tool for identifying and managing public health externalities of large-scale projects [20, 23, 57]. Yet despite established international best practices, no African country has formal regulations that stipulate whether and how public health protection should be incorporated into licensing processes for large-scale mining. In practice, when addressed at all, public health is covered minimally within EIAs, with a focus on environmental determinants of health [12, 15]. As a result, mining companies typically devote limited attention to public health in the absence of a regulatory mandate.

Despite a legal framework built on international best practice, evidence shows that EIA practice in low-income countries delivers weaker results than in industrialised countries. It rarely delivers comprehensive protection, and key environmental and social determinants of public health remain inadequately mitigated, including water, air, and noise pollution, as well as loss of land and livelihoods [1, 2, 33]. The literature consistently points to implementation weaknesses driving outcomes. EIAs are frequently delayed and of uneven quality [4, 14, 61]. public participation remains tokenistic, and, as a consequence, community perspectives are integrated only at the margin. During mine operations, systematic monitoring of the implementation of measures defined in the management plan, a key element of every EIA, is often weak or absent [1, 10, 21, 28, 34, 61]. The literature also points to institutional constraints, including limited budgets, staffing, technical capacity and equipment, compounded by complex procedures and centralised decision-making [35].

Against this background of well-developed EIA regulation delivering weak on-the-ground results, the question arises: what does this mean for strengthening public health protection? Does this make the point of legislating Health Impact Assessment similarly to that of EIA? Or would an international best-practice-inspired HIA regulation experience the same fate as the EIA regulation, which is well-developed but fails to protect the environment effectively? The literature offers only a limited understanding of the policy dynamics that lead states to adopt state-of-the-art regulations while failing to implement them effectively. Many accounts ultimately stop short at the rather uninformative explanation of a ‘lack of political will’ [9].

We address this research gap. We ask what measures and conditions need to be in place for public policies to better protect public health in industrial mining? What political-economy dynamics are hindering or supporting that these conditions are met? To answer this overarching question, we start from the following position: First, Impact Assessments (IA) are widely regarded as the most comprehensive tool for identifying and managing public health externalities of large-scale projects, under conditions of proper implementation [20, 23, 57]. Second, since EIAs and HIAs follow the same conceptual model and intervention logic for managing externalities from large-scale mining projects, much can be learned from analysing the political dynamics that shape EIA implementation, which will be relevant to the health domain. Third, to understand the conditions and dynamics shaping the success and failure of IA implementation in a specific context, the perspectives of actors with firsthand experience implementing IA regulations and their views on what (policy) changes should be made to improve the effective mitigation of environmental and public health risk in large-scale mining are particularly relevant.

**Fig. 1:**
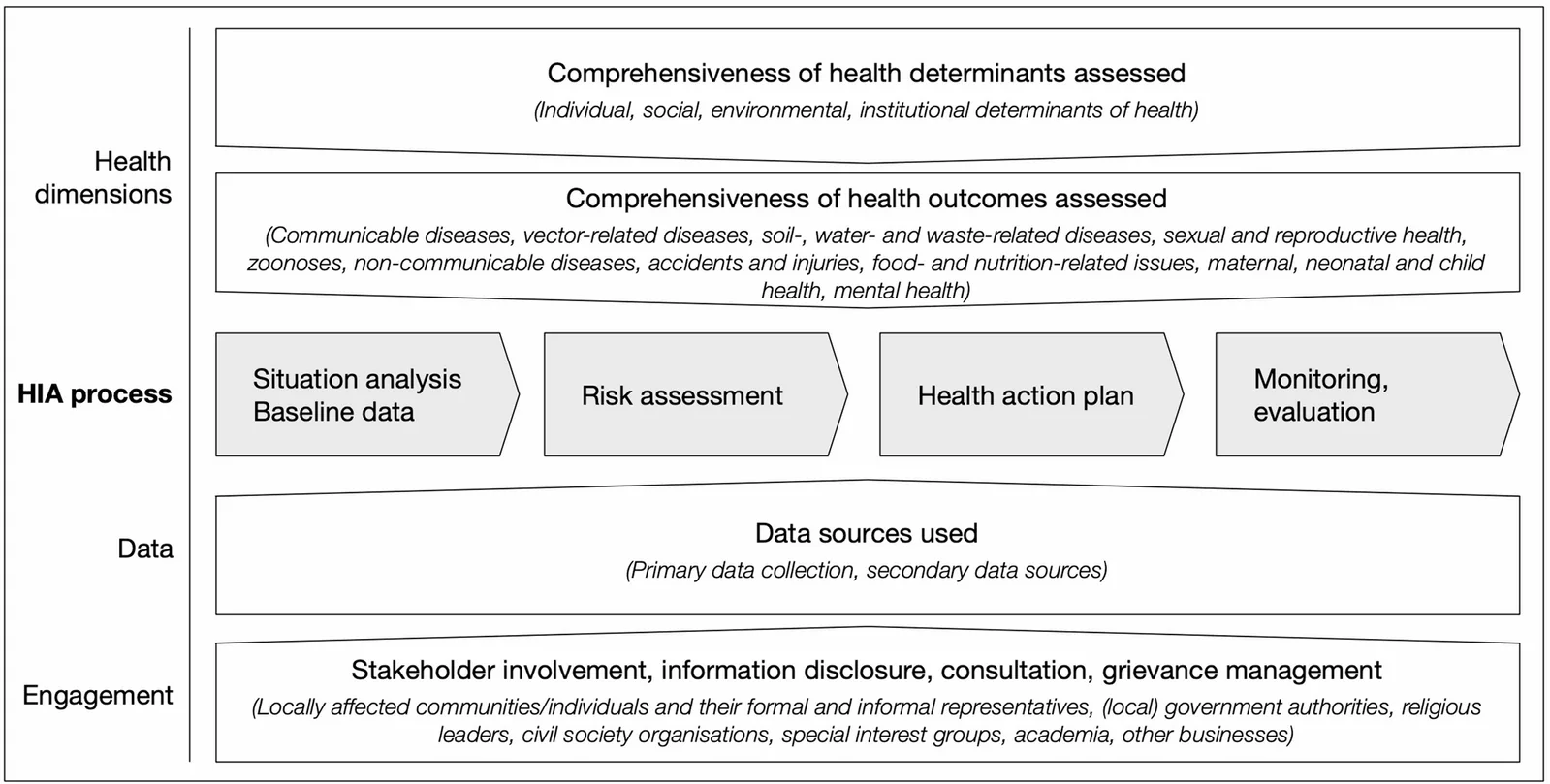
Health impact assessment framework (source: authors, based on 23)

To elicit these views, we unpack the Impact Assessment process into its procedural elements and determinants of quality, using the HIA variant of IA. The proper understanding of the key components of the IA process will inform how the overarching research question can be operationalised. Conducted before project start, an HIA helps licensing authorities and company management understand public health risks and impacts, informing decisions on project design, management and monitoring to safeguard affected populations (Fig. 1). Consistent with IA good practice, IAs start with a *situation analysis* and *baseline data* collection. The resulting information informs the identification and prioritisation of (health) risks by probability and severity. This, in turn, supports the development of a (*health) management plan* that includes measures for prevention, mitigation, and response, as well as assigned responsibilities. Finally, the management plan contains a *surveillance, monitoring and evaluation* strategy to track compliance, detect unanticipated effects and provide early warnings.

(H)IA quality not only depends on duly following this process, but also on (a) how comprehensively it considers – in each step – key health determinants and outcome categories [23, 69]; (b) the quality of data sources and primary data collection; and (c) the level of stakeholder engagement. Key engagement elements include stakeholder identification and analysis, (project) information disclosure, consultation, negotiation and partnerships, grievance management, involvement monitoring, and reporting to stakeholders [23, 24].

Public health regulation in mining can take diverse forms, with mandatory standalone HIA as the most rigorous approach. Alternatively, HIA can be integrated into existing EIA procedures [32, 68]. Advocates of integrated HIA cite better use of limited resources and fewer redundancies [6, 19]. Proponents of standalone HIA argue that, in practice, integrating often leads to omitted or incomplete health analysis [25]. Others consider both options valid if integrated assessments meet the same standards as stand-alone HIA [23]. Strengthening public health in industrial mining, whether through integrated or standalone HIAs, requires political support to prevent direct or indirect resistance to implementation. Identifying stakeholders’ perspectives is essential for understanding the available policy space amid conflicting goals and interest politics [9].

Against the background of the regulatory, procedural, and quality characteristics of IA described in the previous paragraphs, five sets of sub-questions emerge that guide our: *First*, do stakeholders across sectors see any need for policy action? Successful policy development requires support not only from legislators and regulatory institutions, but also from industry and civil society. *Second*, if policy action is needed, which policy options should be prioritised to protect and promote public health? Options include a broad range, including but not limited to integrated versus standalone HIA. Varying emphasis can be put on different elements, such as risk assessment, management plans, review processes, or lifecycle monitoring. Measures can be minimal, extended or comprehensive. *Third*, what level of stringency is preferred? Do stakeholders prefer voluntary measures with incentives or mandatory measures? Is there support for monitoring and enforcement? Should accountability rely on self-regulation or external control? What resources and capacities are stakeholders willing to provide? *Fourth,* how should roles be allocated across public and private actors and within public administration? What should be the roles of mining companies, and what should be the role of the public administration at the national and subnational levels? And what is the relevance of coordination and consultation? *Finally*, what importance is attached to data disclosure?

We address these questions from the perspectives of public-sector, private-sector, and civil-society stakeholders, drawing on their years of experience implementing impact assessments and on what they have identified as critical to enhancing the effectiveness of impact assessments. While we focus on Tanzania, the study forms part of the HIA4SD research programme, a four-country study of public health in industrial mining areas in Burkina Faso, Ghana, Tanzania, and Mozambique [66]. Tanzania has detailed EIA procedures from scoping to monitoring and auditing under the National Environmental Management Council (NEMC). Public health is mentioned briefly in high-level policy documents, but not in the detailed impact assessment regulations. Using Q-methodology to elicit stakeholders’ views and preferences, we find that public, private, and civil society stakeholders recognise the need for reform and prioritise monitoring and compliance with existing provisions over the enactment of new legislation. Main obstacles identified include divergent ministerial priorities and competition between implementation units, and national and subnational administrative levels.

The next section introduces Q-methodology. Section three presents the results, including areas of agreement and disagreement between stakeholders, followed by their interpretation and discussion in section four. Section five concludes.

## Methods

### Study design and rationale for Q-methodology

We employ Q-methodology to uncover how stakeholders understand the underperformance of impact assessments, identify effective solutions, and prioritise measures [8, 39, 70]. Q-methodology is a mixed-methods approach well suited for small-n samples. Participants are asked to rank a set of statements; factor analysis is then used to identify shared patterns in these rankings. These shared patterns are referred to as *perspectives* and represent common viewpoints among participants rather than individual opinions. Together, the perspectives reveal where support, disagreement, and potential coalitions for policy change exist, thereby helping to identify the policy space available for reform [7, 37, 56, 62, 63].

**Fig. 2:**
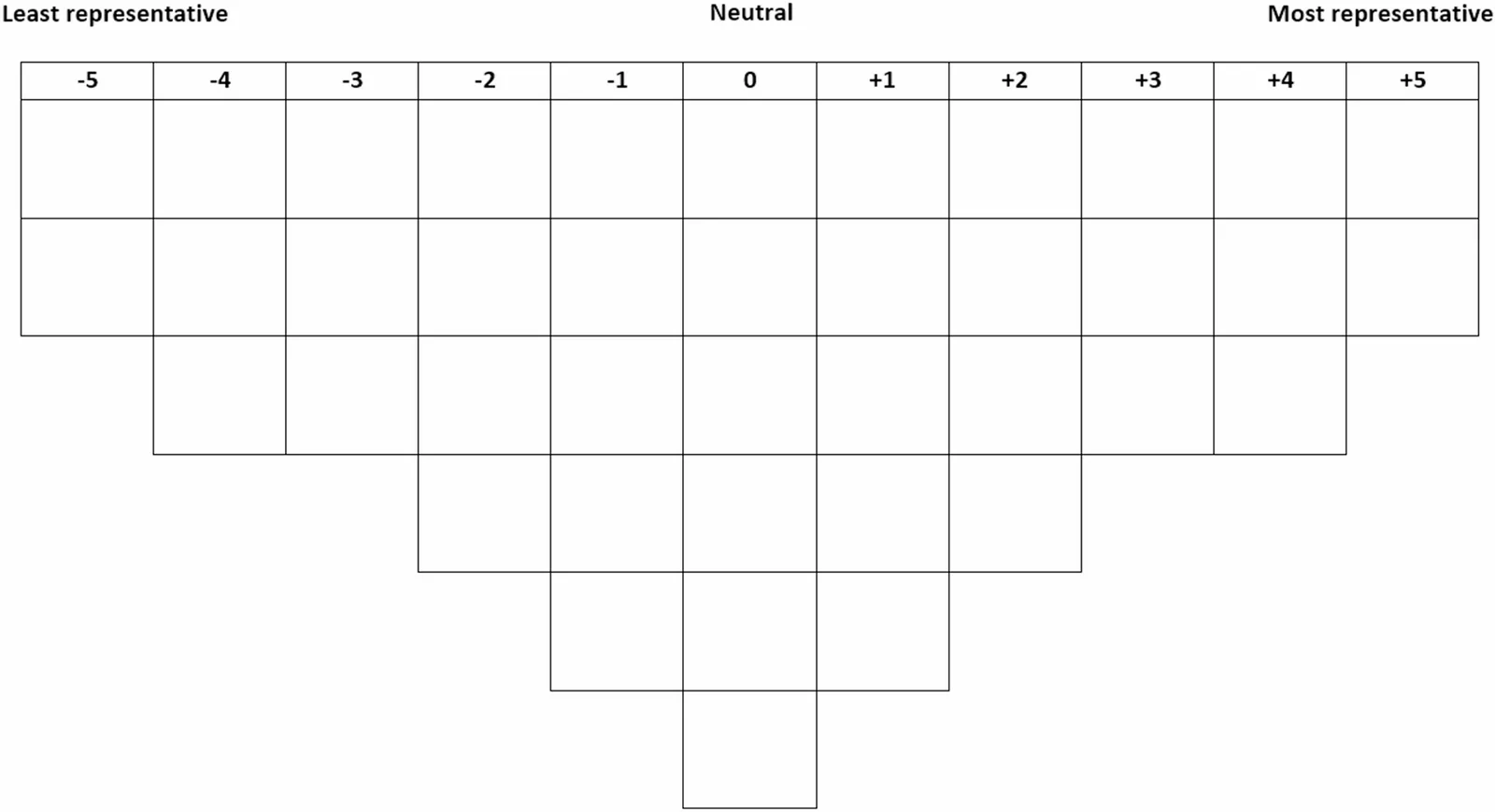
The Q-sorting grid used to rank policy options from most representative (+ 5) to least representative (−5) of participants’ policy preferences (source: authors)

Technically, the statements (Q-set) presented to participants for ranking represent the spectrum and arguments that characterise a policy debate, in our case, the implementation of impact assessments to protect public health [62, 63]. Participants sort these statements according to their level of agreement. To elicit clear distinctions, the sorting grid presented to participants follows a forced normal distribution, as shown in Fig. 2 [64]. The sorted statements from a participant are called Q-sort. The Q-sorts from all study participants are analysed using factor analysis to identify groups of participants who have ranked the statements in a similar way. Unlike conventional factor analysis, which examines relationships between variables across respondents, Q-methodology applies *by-person factor analysis*, correlating participants with one another based on their complete Q-sorts. Participants whose rankings are highly similar load on the same factor, which is then interpreted as a shared perspective. The factor arrays generated from these shared ranking patterns provide an idealised representation of each perspective and form the basis for qualitative interpretation. [3, 7, 56, 63].

Prior Q-studies in extractive sector policy have examined agreement and disagreement on environmental policy in Ghana’s gold mining sector [58], stakeholders’ experiences in EIA public consultation in South Africa [54], community perceptions of mining impacts on local development in Vietnam [43], Rwanda [65] and Ecuador [60], and conflicts over land use for rare earth mining in the US [27].

### Concourse and Q-set development

A critical step in Q-methodology is the construction of the Q-set since the validity of a Q-study depends on the extent to which the Q-set represents the breadth of perspectives and policy options present in the discourse [37, 64]. The Tanzania Q-set was developed collaboratively by researchers from the four-country HIA4SD research programme [66] to enable cross-country comparison of regulatory issues and reform options [48–50].

To ensure broad thematic coverage when developing the Q-set, we used the following sources: First, we reviewed the ‘concourse’, meaning academic and policy literature on public health impacts of large-scale mining, the role of impact assessment, and the political economy of mining regulation [7, 62, 63]. Second, we reviewed the regulatory and institutional frameworks governing large-scale mining in Tanzania and the other HIA4SD countries. Third, we conducted expert interviews with representatives from the ministries of mining, health and environment, the private sector and civil society in all four countries. Interviewees were purposefully selected for expertise in mining governance and impact assessment practice. Fourth, we drew on focus group discussions at mining sites across all four countries. In Tanzania, focus groups were conducted in 2019 in Bulyanhulu, Buzwagi, and Geita. Participants included local health service representatives and community members near industrial mining sites.

Initial statements were crafted inductively from the concourse and refined using the HIA quality dimensions outlined above [7, 63, 64]. Statements were framed as policy options varying in scope, stringency, responsibilities, accountability and coordination. The resulting 234 initial statements were later condensed into a sample of 40. A pilot was conducted with stakeholder representatives to ensure comprehensibility and topical coverage. The pilot results confirmed that the statements adequately represent the concourse and helped to fine-tune the wording of several statements to improve clarity. The revised final Q-set comprised *n* = 40 statements (see Table 1). The original Q-set was developed in English. For Tanzania, statements were administered in Swahili (Appendix A, Table A.2). Further details and the original publication of the Q-set are provided in [48].

**Table 1:** Factor Q-scores for each item in the Q-sample

*No*	*Q-statement*	*F1*	*F2*	*F3a*
		Q-score	Q-score	Q-score
S1	The existing EIA are sufficient. No need for additional measures for public health	−3	−2	+ 1
S2	Mining companies shall decide on their own how they want to address public health issues	−4	−3	−5
S3	Requiring a separate HIA would overburden mining companies and deter investment	−4	−2	−4
S4	With the current EIA requirements, companies are free to include public health considerations where relevant. This is sufficient	−4	−4	−4
S5	EIA requirements need to be revised to include comprehensive consideration of public health	−2	−2	−2
S6	Requiring a separate HIA would overburden the licensing authorities and create additional bureaucratic barriers	−3	−3	−1
S7	The government must require from mining companies a stand-alone HIA (in addition to the EIA) to mitigate adverse health impacts	−3	+ 1	−1
S8	Mining licenses must not be granted or renewed without a "public health management plan"	+ 3	−1	+ 2
S9	Build up technical capacity in the NEMC to assess the quality of public health aspects in EIAs	+ 1	−2	+ 1
S10	Build up technical capacity in the MoH to assess the quality of public health aspects in impact assessments	+ 4	−3	+ 2
S11	Baseline data on health indicators must be collected to assess the health impacts of mining projects	+ 5	−1	−1
S12	The MoH must provide companies with clear methodological guidelines for conducting HIA	+ 2	−1	−4
S13	Monitoring and technical staff must have protected tenure to prevent political appointments and shield regulatory institutions from political interference	−1	−2	+ 2
S14	A separate department needs to be created within the MoH to monitor the health impacts of large-scale mining projects	−1	−4	0
S15	Mining companies can voluntarily disclose information on health impacts to local communities	−5	−5	−3
S16	The MoH requires more financial resources to monitor public health management of mining companies	+ 2	−4	+ 4
S17	The MoH must publicly disclose annually the results from public health monitoring in mining areas	+ 3	−1	+ 1
S18	Only regular audits and inspections by public officials can ensure that mining companies adhere to public health protection measures	0	0	−3
S19	The government must amend mining regulations to strengthen the monitoring of mining companies’ health performance	+ 1	0	−2
S20	Monitoring of public health impacts should be left to mining companies which can publish annual monitoring reports	−5	−5	+ 3
S21	Capacity to conduct smaller public health monitoring should be build up at district level and within regional administrations	0	0	−2
S22	The MoH needs to be directly involved in regular mining-site inspections and the measurement of pollutants	+ 1	0	−5
S23	The government must establish a clear sanctions framework to penalize non-compliance with public health regulations and standards	+ 5	+ 2	+ 1
S24	When mining projects are decommissioned, mining companies must present rehabilitation plans that include targeted measures that address public health	+ 1	+ 1	−3
S25	Create a formalized forum for stakeholders from government, civil society, academia, and the private sector to ensure continuous discussion of health challenges related to mining	0	0	+ 3
S26	Establish a centre of expertise at a university or research institution to train civil servants in HIA	+ 2	1	−2
S27	Coordination between actors responsible for issuing licenses, and those responsible for monitoring must be greatly intensified	−2	+ 2	0
S28	The MoH must have a formal role in the licensing process to ensure the inclusion of HIA in current EIA	0	0	0
S29	The MoH must publish its technical advice on impact assessments to avoid being disregarded during the final decision-making process	−1	+ 2	+ 4
S30	Strengthen the technical commission in which all actors responsible for issuing licenses coordinate (health, environment etc.) so that their findings cannot be overruled	0	+ 4	+ 2
S31	At the beginning of each impact assessment, companies must carry out consultations on health issues with local health professionals, women and civil society groups, and communities	+ 1	+ 4	0
S32	Companies must share the results of their HIA in a simplified format to make it intelligible for ordinary citizens and illiterate community members	0	+ 3	+ 1
S33	The government is responsible to invest in public health services in mining areas to provide equipment, medicines, and training for health personnel	+ 2	+ 1	0
S34	The government must establish a platform for coordination between the local health system and mining companies to ensure that voluntary health projects of companies benefit local communities	+ 3	+ 3	+ 4
S35	Mining companies should be encouraged to communicate an annual application window for corporate social responsibility projects so that health system actors can submit proposals	−2	+ 4	−1
S36	Mining companies shall be required to finance regular health monitoring surveys in mining areas	+ 4	+ 2	+ 3
S37	Mining companies shall be obliged to conduct regular awareness campaigns on (mining related) health risks and promote best health practices	−1	+ 5	+ 5
S38	Mining companies must be obliged to finance goods and equipment to local health services, including ambulances and a medical laboratory for health testing	+ 4	+ 3	0
S39	Mining companies shall be required to run a health facility open to the public	−1	+ 1	+ 5
S40	Mining companies should publicly communicate their responses to (health-related) grievances and complaints	−1	+ 5	−1

Source: authors; Q-statements were first published in [48]

### Participant selection

Participants were purposefully selected based on the following criteria: being policy stakeholders and having an in-depth understanding of and experience with Tanzania’s public health, environmental and/or mining regulatory frameworks, including impact assessment. This ensured that Q-statements were comprehensible and meaningful to participants [63]. We also ensured diversity in backgrounds and professional roles to maximise the diversity of engagement with impact assessment processes [43, 58, 62]. In total, 23 participants (*P* = 23) from government, the private sector, civil society and academia were included (Table 2).^1^

**Table 2:** Stakeholder and selected participants

Stakeholder		
Public sector (*P* = 16)	Private sector (*P* = 4)	Civil society (*P* = 3)
National level (*P* = *10)*	▪ Industrial mining company (*P* = 1) ▪ Tanzania Chamber of Mines (*P* = 1) ▪ Private consultancy firm I (*P* = 1) ▪ Private consultancy firm II (*P* = 1)	▪ Local civil society organization (*P* = 1) ▪ Local representation of international development NGO (*P* = 1) ▪ Local academia member (*P* = 1)
▪ Ministry of Mines (MoM) (*P* = 4) ▪ Ministry of Health (MoH) (*P* = 2) ▪ National Environmental Management Council (NEMC) (*P* = 2) ▪ President’s Office, Regional Administration and Local Government (PO-RALG) (*P* = 2)		
Subnational level (*P* = 6)		
▪ PO-RALG, district-level health and environmental management professionals (*P* = 4) ▪ NEMC, zonal managers (*P* = 2)		

Source: authors

### Q-sorting procedure

Data collection involved participants completing Q-sorts in individual face-to-face sessions, followed by brief qualitative interviews. Participants were contacted in advance and informed about the study background and purpose. All participants provided informed consent.

Participants were instructed to rank the 40 policy statements in two steps, based on perceived importance and effectiveness. First, they sorted statements into three piles: most representative, least representative, and neutral. They then placed all statements on the Q-sorting grid from + 5 (most representative) to −5 (least representative), with 0 as neutral *(*Fig. 2*)*. Participants could adjust rankings until they were satisfied with the final distribution. After sorting, participants explained their ranking choices, especially for statements placed at + 5 and −5. These explanations supported factor interpretation and helped clarify stakeholder viewpoints.

### Data analysis

In Q-methodology, factor analysis identifies patterns in how participants rank statements and groups individuals with similar viewpoints. This translates the raw sorting data into interpretable factors representing distinct shared perspectives.

**Fig. 3:**
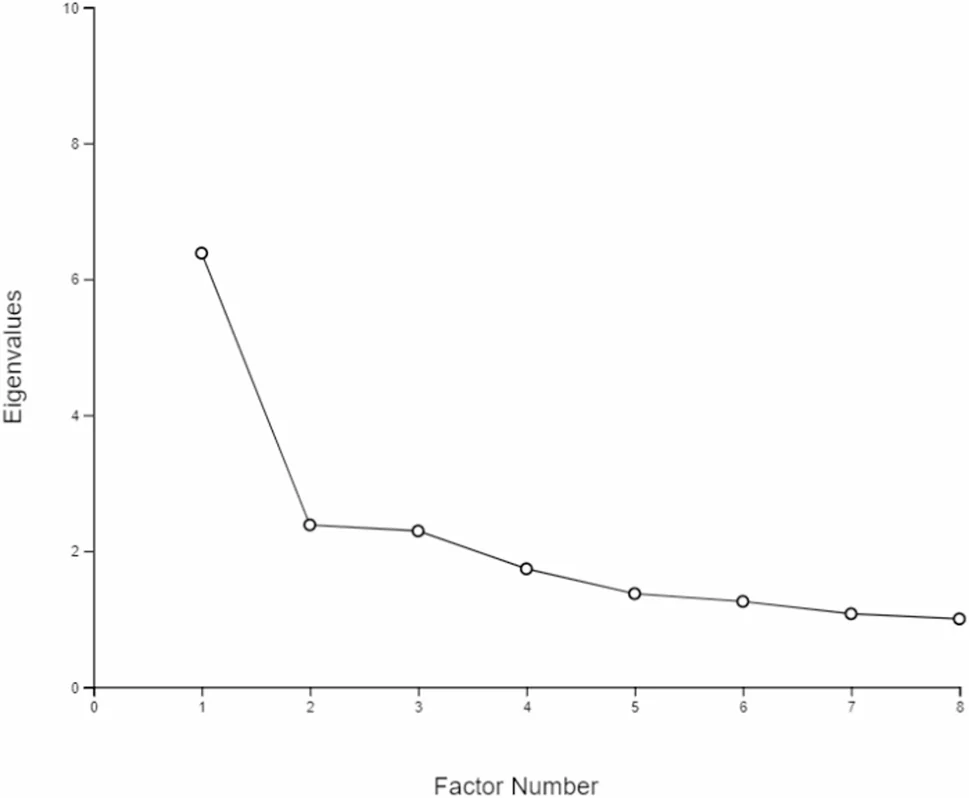
Scree plot (source: authors)

We analysed 23 Q-sorts, using KADE (V1.2.1) software. We initially extracted eight factors and rotated three using Varimax. Factor selection draws on statistical criteria and theoretical considerations. However, since there are no definitive rules on the number of factors to select, the process remains a matter of expert judgment [7, 63, 64, 70]. We selected a three-factor solution based on theoretical relevance and commonly accepted statistical criteria in Q-methodology: the Kaiser-Guttmann criterion, the significant loading test, the minimum variance test (minimum factor variance > 8% and cumulative variance > 30%); and the scree test, which shows two slope changes, after Factors 2 and 3 (Fig. 3) [5, 63].

Factor loadings of 0.41 or higher were considered significant at *p* > 0.01. Of 23 Q-sorts, 20 loaded significantly on at least one factor, with eight loading on Factor 1 (F1), seven on Factor (F2), and three on Factor 3 (F3). Two Q-sorts were confounded, loading significantly on more than one factor. We included non-confounded Q-sorts and excluded confounded Q-sorts and null cases [63, 64].

Together, the three factors explain 48% of the variance, with 18% attributable to F1, 20% to F2, and 10% to F3. Correlations between factor scores are insignificant; further factor characteristics are reported in Appendix A, *Table A. 2*. For F3, one Q-sort loads positively and two load negatively, rendering the factor bipolar. For interpretation, we inverted F3 and split it into F3a and F3b, yielding two positive loadings and one negative loading. F3a is a factor array based on two Q-sorts (TZ16, TZ19), whereas F3b corresponds to a single Q-sort (TZ09). Table 1 reports Q-scores for each statement for the factor arrays of F1, F2, and F3a.

Factor interpretation and the development of the perspectives started with the identification of patterns at the level of the main categories defined in the research questions (the need for policy action; prioritisation of policy options; preferred stringency of policy measures; roles and responsibilities across public and private actors and within public administration; the importance attached to data disclosure) and verified with topical experts from the research team. The rich information gathered during the interviews helped to contextualise and refine the perspectives.

### Reporting standard

Reporting standards for Q-methodology studies are only emerging, and no commonly accepted standard exists [11, 13]. Reporting of this study followed the Q-methodology reporting standard proposed by Dieteren et al [13]. A completed reporting checklist is provided with the supplementary material.

## Results

Each retained factor represents a distinct perspective on which policy measures best promote public health in large-scale mining. As mentioned earlier, the factors are referred to as *Perspectives*.

### Shared positions across the three perspectives

All three perspectives agree that the current EIA framework does not sufficiently account for public health impacts (S1). They also reject the claim that implementing HIA would impose excessive burdens on mining companies, discourage investment (S4), or create additional bureaucratic obstacles for licensing authorities (S6). This suggests that introducing HIAs is both feasible and desirable from their standpoint.

However, this does not translate into broad support for mandatory standalone HIA (S7) or comprehensive incorporation of public health into EIA (S5). Both options receive negative Q-scores across all three perspectives. But this does not necessarily indicate resistance to regulatory change, as questions about the legal framework are often eclipsed by other concerns. Among those emphasising which impacts should be legally regulated, preferences diverge between perspectives, and no consistent pattern emerges.

### Perspective 1: The compliance perspective – to protect public health, strengthen public monitoring and oversight, and impose sanctions

In short, the *Compliance Perspective* sees public health protection primarily as a matter of compliance and enforcement within the existing legal framework. It emphasises the importance of robust baseline data, clearly defined health management requirements, stronger public-sector monitoring, and sanctions for non-compliance, while holding mining companies financially responsible for monitoring and supporting local health services.

*Perspective 1* reflects a cross-cutting view across stakeholder groups except civil society: representatives of all public sector units at the national and sub-national level included in the study (MoH, MoM, NEMC, PO-RALG) and private sector representatives load significantly on this perspective. Taken together, the policies prioritised in the *Compliance Perspective* reveal a coherent strategy focused on the implementation of public health protection. They can be clustered around three pillars meant to improve compliance. The foundational pillar is baseline data collection for public health indicators (S11, + 5), an indispensable precondition for improved monitoring, as a senior MoH official noted: “It is important to ensure that you have the baseline data before [the start of mining operations] in order to understand the effect after any intervention is done to people surrounding mining sites” (TZ01). This call for a robust baseline is paired with a shared demand for a ‘public health management plan’ as a licensing requirement (S8, + 3). In impact assessment, the management plan details the binding measures to avoid, mitigate and manage risks and defines operational indicators and benchmarks that enable monitoring and enforcement.

The second pillar concerns responsibility for monitoring public health impacts. Proponents of the *Compliance Perspective* reject transferring monitoring to companies (S20, −5; S15, −5) and entrust this function to the public sector. They also support the annual publication of public health monitoring results in mining areas (S17, + 3). While companies should bear monitoring costs (S36, + 4), the technical capacity of the MoH should be strengthened (S10, + 4) and, as a high-ranking MoM official stressed, “the Ministry of Health would necessarily require more financial support to monitor public health management of mining companies” (TZ26). Interesting to note is that NEMC officials within *Perspective 1* do not endorse this proposal (TZ02, −1; TZ25, −4), indicating resistance from the entity in charge of environmental monitoring.

The third pillar of the *Compliance Perspective* is a sanctions framework to enforce compliance with public health regulations and standards (S23, + 5). Strong support for sanctions comes from regional-level public officials from PO-RALG (TZ11, + 5; TZ07, + 4) and NEMC (TZ02, + 4), who argue that “imposing fines on mining companies that do not adhere to government guidelines is important to strengthen public health control” (TZ11). However, the MoM representative signals reservations towards sanctions (TZ26, −3).

Finally, *Perspective 1* requires mining companies not only to pay for monitoring (S36, + 4) but also to finance goods and equipment for local health services (S38, + 4). As we will see next, this links *Perspective 1* to *Perspective 2,* which holds that mining companies have a corporate responsibility to contribute financially and materially to health services in mining areas.

### Perspective 2: The local perspective – no protection of public health without local actors in the lead

In short, the *Local Perspective* argues that protecting public health in mining areas requires strong local leadership. It emphasises prevention, community engagement, and corporate accountability, and locates responsibility for monitoring at the regional and district levels rather than with companies or central government institutions. The underlying view is that local actors are best placed to understand, monitor, and respond to mining-related health risks.

*Perspective 2* can be labelled the *Local Perspective* as it is primarily shaped by representatives closely connected to the mining areas who experience the day-to-day mining operations firsthand. It includes district-level PO-RALG and MoM officials, as well as representatives from a development NGO, a mining company and a consultancy.

The strategy advanced by *Perspective 2* pivots on strengthening subnational and local-level actors. Three complementary priorities emerge. First, it prioritises preventive and curative public health measures. Mining companies should be required to run regular awareness campaigns on mining-related health risks and promote good health practices (S37, + 5). Participants viewed this as a company responsibility and an effective, low-cost way to help communities understand health risks and take preventive action (PO-RALG, TZ07; NGO, TZ23). *Perspective 2* further prioritises mandatory company support for local health services, including equipment such as laboratory facilities and ambulances (S38, + 3). This support should be coordinated through a government-led platform that brings together mining companies and local health providers, rather than being left to company discretion (S34, + 3). Companies should open an annual application window so that health providers can apply for support (S35, + 4). As one participant noted, “where there are negative impacts resulting from mining activities, there should be curative services, and this has to be part of their corporate social responsibility” (PO-RALG, TZ07; similarly: local MoM, TZ10).

*Perspective 2* also places strong emphasis on accountability to local stakeholders. Participants argued that mining companies should consult local health professionals, women, civil society groups, and communities on health issues at the start of each impact assessment (S31, + 4). They also favoured making impact assessment results available in a simple format so that all community members, including those with limited literacy, can understand the identified risks and proposed measures (S32, + 3). In addition, companies should publicly respond to health-related grievances and complaints (S40, + 5). As a district-level PO-RALG environmental management official explained, “it is important to have truth and openness around the investments done by mining companies” (TZ12) to address public dissatisfaction and local grievances effectively.

The third core element of *Perspective 2* is monitoring by local public authorities. Participants rejected leaving public health monitoring to mining companies, whether through annual reports (S20, −5) or voluntary disclosure to communities (S15, −5). They also opposed concentrating additional monitoring resources and responsibilities at the central Ministry of Health level (S16, −4; S14, −4). Instead, they argued that monitoring should be carried out by regional and district-level authorities because “the companies themselves cannot do monitoring, but control has to be done externally” (PO-RALG, TZ07) and because companies are primarily driven by profit rather than public health concerns (PO-RALG, TZ12). As one participant explained, “the task to monitor the health impacts of large-scale mining projects should be delegated to regional medical offices for close follow-up” (MoM, TZ12). Another argued that “funds should be brought to regional and local governments who have the core responsibility of monitoring public health in respective mining areas” (PO-RALG, TZ12).

*Perspective 2*, therefore, reflects scepticism about the ability of central government institutions to monitor health impacts effectively at the local level. Instead, participants saw the central government’s role as defending public health concerns when negotiating the terms and conditions of mining licenses. Accordingly, they supported strengthening the Technical Advisory Committee so that its recommendations cannot be overruled (S30, + 4). Composed of experts in mining, labour, environmental and public health issues, it evaluates impact assessments and recommends design adaptations and the terms of management plans [18, 42]. Participants’ comments suggest concern that its recommendations have at times been disregarded during licensing decisions. This concern about the MoH’s limited weight in the MoM-led licensing process links *Perspective 2* to *Perspective 3.*

### Perspective 3: The partnership perspective – strengthening the ministry of health and corporate-led public health management

In short, the *Partnership Perspective* advocates a more proactive role for mining companies in protecting public health, combined with stronger guidance and oversight from the Ministry of Health. It emphasises company-led health services, awareness raising, and collaboration with local health actors, while supporting clearer public health guidance within the impact assessment process.

*Perspective 3* is defined by two Q-sorts from a senior public health official at PO-RALG (TZ16) and a member of academia (TZ21), corresponding to Factor 3a. Given the limited number of defining Q-sorts, this perspective should be interpreted with caution. Nevertheless, it reveals identifiable tendencies that provide useful reference points for further discussion.

A defining feature of *Perspective 3* is its support for a more active role for mining companies in protecting public health. Participants strongly favoured requiring companies to operate a public health facility (S39, + 5) and run regular awareness campaigns on mining-related health risks (S37, + 5). They also supported a coordination platform between mining companies and local health providers to ensure that voluntary health projects benefit surrounding communities (S34, + 4). At the same time, they argued that the Ministry of Health (MoH) should receive additional resources to monitor how companies manage public health in mining areas (S16, + 4).

*Perspective 3* also strongly supports the publication of MoH technical advice on impact assessments (S29, + 4). Participants viewed this as a practical way to raise the profile of public health concerns, support project proponents in integrating health issues into EIAs, and assist the Technical Advisory Committee and NEMC in evaluating submissions. Unlike legal reforms, a technical note can be issued and updated by the Ministry without legislative changes. While it does not carry the same legal weight as an ordinance or law, it clarifies the administration’s interpretation of existing legislation and reduces uncertainty for actors confronted with broadly formulated legal requirements, as observed in Tanzania. At the same time, *Perspective 3* rejects prescribing detailed methodological guidelines for impact assessment (S12, − 4), preferring guidance without extensive procedural requirements and micromanagement.

*Perspective 3* is contrasted by the view of a senior Ministry of Mines official (Factor 3b), who strongly opposed requiring mining companies to operate public health facilities (S39, − 5). In the official’s words, “the management of public health is purely a government role that can’t be easily transferred to mining companies” (TZ09). The participant showed only limited support for company-led awareness campaigns (S37, + 1) and additional MoH resources for monitoring (S16, + 1). However, the official strongly supported the publication of MoH technical advice on impact assessments (S29, + 5), describing it as “more practical to be implemented to promote inclusion of public health issues in the EIA process” (TZ09). The participant also favoured revising EIA requirements to explicitly include public health considerations (S5, + 4).

## Discussion

What policy space do the three perspectives reveal for advancing public health in mining areas in Tanzania? We assess the three perspectives against the five research questions to clarify the available policy space (Table 3).

**Table 3:** Policy space for strengthening public health in mining areas in Tanzania, along the five sets of research questions, complemented withrelevant tensions observed between stakeholders

	Perspective 1: Compliance View	Perspective 2: Local View	Perspective 3: Corporate-led health management (3a vs. 3b)
	8 Q-sorts from MoH, MoM, NEMC, PO-RALG, company, consultancy firm; absent: civil society	7 Q-sorts from (local PO-RALG, NGO, MoM; absent: MoH, NEMC	3a: 2 Q-sorts from PO-RALG, local academia 3b: 1 Q-sort from MoM only
**Need for Action**			
Current regulation	EIA regulations are insufficient	EIA regulations are insufficient	EIA regulations are insufficient
Reasonable request	HIA would not deter investment and not overburden companies/administration	HIA would not deter investment and not overburden companies/administration	HIA would not deter investment and not overburden companies/administration
**Priority areas**			
Broad legal reform	HIA and EIA reforms are rejected	Indifference towards HIA and EIA reforms	3a: indifference 3b: support for EIA reform
Strengthen specific institutions	Management plan on public health	– –	MoH to issue technical note on public health management
Health service provision	1: Companies to provide goods/equipment for public health services 2:– – 3:– –	1: Companies to provide goods/equipment for public health services; application window for CSR projects 2: Companies to conduct awareness campaigns 3:– –	1:– – 2: 3a: Companies to conduct awareness campaigns 3: 3a: Companies to run health facility (opposed by 3b)
**Stringency**			
Voluntary/self-monitoring	1: Monitoring not to be left to companies’ discretion 2:– –	1: Monitoring not to be left to companies’ discretion 2:– –	1: Monitoring not to be left to companies’ discretion. 2: Industry self-regulation rejected
Mandatory monitoring	Yes	Yes	Yes
Enforcement	Sanctions framework	– –	– –
**Actor roles and responsibilities**			
Companies vs. MoH National (N) vs. MoH sub-national (SN)	1: MoH-N to lead monitoring and enforcement; companies to provide funding for monitoring 2: MoH to provide health services	1: MoH-SN to lead for monitoring; cooperation with companies 2: Public-private cooperation in health promotion	1: MoH-N to lead monitoring 2: Public-private cooperation in health promotion (and services)
Expand capacities	Knowledge and financial resources for MoH-N (no support from NEMC)	Technical expertise, staff and funding for MoH -SN	3a: Financial resources for MoH-N
Decision power	– –	Strengthening Technical Advisory Committee so it can’t be overruled in decision making	Issuing Technical Note to strengthen MoH position in licensing process
**Cooperation **between actors			
Coordination	– –	LOCAL platform for coordination between the local health system and Companies	3b: NATIONAL platform for govt, civil society, academia, private sector
Inclusion/consultation	– –	Companies to consult with local health professionals, communities, women and civil society for EIA	3b: Companies to consult with local health professionals, communities, women and civil society for EIA
**Transparency**			
Public disclosure	Regular disclosure of monitoring results	– –	– –
Company disclosure	Mandatory also for companies to inform about negative impacts	publish responses to (health-related) grievances and complaints	– –
**Conflicts **			
	1: NEMC vs MoH over responsibility and financial resources for monitoring 2: MoM resists strong sanctions 3:– –	1: MoH-N vs MoH-SN over responsibility for monitoring 2:– – 3:– –	1:– – 2:– – 3: 3b: MoM rejects that mining companies should run health facilities open to the public

source: authors

### Need for reform

All three perspectives agree that protecting and promoting public health requires policy attention and will not overburden mining companies. Hence, all stakeholders support placing the topic on the political agenda and initiating a policy process.

### Priority measures and their stringency

Stakeholders show little appetite for sweeping legal reforms, such as drafting HIA or EIA legislation, possibly reflecting dissatisfaction with earlier mining reforms that generated political attention without substantial change [26, 46, 47]. Instead, participants prioritise specific measures within the existing regulatory setup.

Compliance, from baseline data collection to penalties, is a shared priority across all three perspectives. They also agree that monitoring cannot be left to companies but must be a core task of the MoH. This focus echoes longstanding implementation and enforcement challenges, including limited accountability in enforcing the Environmental Management Act regarding EIAs in Tanzania [36, 55]. This may explain why sector stakeholders prioritise strengthening implementation over creating new laws.

The second priority concerns direct provision of health services at the mine-site level. Measures range from raising awareness and promoting good health practices to requiring companies to contribute to public clinics and, in some cases, mandating that company-run clinics be open to the local population. However, perspectives differ on which measures should be prioritised and whether running public clinics should be the responsibility of a mining company.

### Actors

A central issue is the distribution of tasks and responsibilities between mining companies and the MoH. Overall, participants support a stronger role for the MoH across all three perspectives. They do not want to vest public health monitoring responsibility in private companies. However, a closer look reveals several differences that complicate the distribution of responsibilities within the public sector. While *Perspectives 1* and *3* refer to the MoH at the central level, the *Local Perspective* advocates strengthening the role of subnational-level actors within the public health system. These preferences reflect a longstanding tension between national and subnational government roles. Tanzania has pursued decentralisation for more than 20 years. Nevertheless, the central government retains tight control over subnational room for manoeuvre, including through centralised fund allocation [29–31, 45]. The *Local Perspective* calls not only for shifting monitoring responsibility to the subnational level but also for capacity building and the transfer of funds. A second tension arises between NEMC and the MoH, as NEMC does not support proposals to strengthen the MoH’s technical capacity or provide it with additional resources for monitoring. Since NEMC currently leads environmental monitoring, this position may reflect a preference for maintaining a centralised monitoring system on efficiency grounds or concerns about redistributing resources, responsibilities, and institutional influence to the MoH. A third point of contention concerns monitoring and enforcement. While actors from the environment and health sectors favour stronger monitoring, compliance controls, and sanctions, the Ministry of Mines (MoM) prefers a more lenient approach to enforcement.

Beyond this inter-institutional tussle, concerns persist about the weak position of institutions tasked with protecting environmental and social concerns in the licensing process. The institutional power dynamics in the licensing decision process have been analysed in the literature, finding NEMC in a particularly weak position. As a result, NEMC may refrain from unpopular decisions to avoid being seen as anti-development, undermining its *de iure* independence as the environmental regulator [55]. Study participants repeatedly referred to such cases. This may explain why proposals to strengthen the Technical Advisory Committee, also called the Technical Review Committee, feature prominently, aiming to prevent its recommendations from being overruled. Other measures point in the same direction, including MoH technical notes on addressing public health in EIAs and measures to strengthen management plans. These measures are expected to advance public health and bolster the institutional position of environmental and public health interests in licensing.

### Coordination and inclusion

Proposals for coordination platforms at the national level (F3b) and the local level (*Perspective 2*) gather limited momentum. The same holds for consultation with local health professionals and communities during EIAs, which only the *Local Perspective* prioritises. Community inclusion in assessment and decision-making often remains a tick-box exercise rather than genuine engagement, leaving communities dissatisfied and frustrated [36, 40].

### Transparency

The *Compliance Perspective* calls for disclosure of monitoring results as part of a compliance strategy. The *Local Perspective* also calls for local transparency, requiring companies to publish responses to (health-related) grievances to strengthen accountability to host communities. As with coordination platforms, transparency attracts limited momentum. This aligns with research on social accountability, which shows that transparency alone does not advance accountability [16, 17].

Future research could deepen the understanding of de facto implementation, governance performance, bureaucratic incentives, and political economy dynamics. More detailed implementation studies are needed on the detail and stringency of EIA and HIA management plans; how monitoring in mining areas is implemented in practice; health data infrastructure near mining sites; and enforcement gaps where compliance breaks down, including inspection capacity, political interference, funding, and data systems. Another research area is bureaucratic politics, including ministry incentives across health, mining, environment, and finance; budget allocation patterns; and the political economy of enforcement, including local elites, revenue dependence, and regulatory capture. Comparative cross-country research could identify institutional designs associated with stronger monitoring outcomes.

## Conclusions

This study identifies a pathway to strengthen public health protection in Tanzania’s industrial mining sector that appears to enjoy support across stakeholder groups. The identified policy space offers a promising basis for reform. Across stakeholder groups, there is broad agreement that the principal challenge is not the absence of regulation but weaknesses in implementation. The study suggests that reform efforts should focus on improving the performance of existing institutions and procedures rather than expanding the regulatory framework. For policymakers and development partners, strengthening implementation capacity, inter-agency coordination, and local-level monitoring may yield greater public health gains than pursuing comprehensive legislative reform.

The findings point to five priority areas for policy action: (1) systematic collection of baseline health data in mining areas; (2) stronger consultation and community engagement throughout the project cycle; (3) regular public-health monitoring accompanied by transparent publication of results; (4) strengthened grievance and accountability mechanisms; and (5) closer collaboration between mining companies and local health systems to improve service provision. In addition, clearer guidance from the Ministry of Health and stronger coordination between health, mining and environmental authorities could address institutional fragmentation and improve implementation.

A final implementation priority concerns addressing tensions among ministries responsible for health, mining, and environmental governance, whose mandates, incentives, and priorities do not always align. Successful reform is likely to depend not only on technical design but also on the ability to reconcile competing institutional interests and develop credible arrangements that provide tangible benefits across participating agencies and administrative levels. Without such accommodation, implementation may, again, be slowed by bureaucratic competition, selective compliance or limited cooperation.

## Supplementary Information

Supplementary Material 1.

Supplementary Material 2.

Supplementary Material 3.

Supplementary Material 4.

Supplementary Material 5.

Supplementary Material 6.

## Data Availability

All data are available as supplementary data files.

## Abbreviations

EIA: Environmental impact assessment
HIA: Health impact assessment
MoH: Ministry of Health
MoM: Ministry of Mining
NEMC: National Environmental Management Council
NGO: Non-governmental organisation
PO-RALG: President’s Office – Regional Administration and Local Government
UDSM: University of Dar es Salaam

## Footnotes

1 A full list of study participants (anonymized) is presented in Annex A, *Table A.3.*
