# Supplementary material for: Protecting public health in mining: a Q-methodology examination of policy space and reform pathways in Tanzania
Source: BMC Public Health. 2026 Jul 27;26:3036. doi: 10.1186/s12889-026-28398-7 (PMC13688855; doi:10.1186/s12889-026-28398-7)
Supplement: Supplementary file 1 [file 12889_2026_28398_MOESM1_ESM.docx]

## Table A.1: Q-sample items in Swahili

| *No.* | *Q-statement* |  |  |  |
| --- | --- | --- | --- | --- |
| S1 | Upembuzi wa kimazingira uliopo unajitosheleza. Hakuna tena haja ya kuchukua hatua za ziada kwa ajili ya afya ya jamii. | | | |
| S2 | Kampuni za uchimbaji madini zitachagua zenyewe namna ya kushughulikia masuala ya afya ya jamii. | | | |
| S3 | Hitaji la kufanya upembuzi unaojitegemea wa masuala ya afya utaongezea mzigo mzito kwa makampuni ya madini na kupunguza uwekezaji. | | | |
| S4 | Kwa mahitaji ya sasa ya kufanya upembuzi wa kimazingira makampuni ya madini yako huru kujumuisha masuala ya afya ya jamii pale panapohusu. Hiyo inatosha. | | | |
| S5 | Mahitaji ya kufanya upembuzi wa kimazingira yanahitaji kurejewa ili yajumuishe masuala afya ya jamii. | | | |
| S6 | Upembuzi unaojitegemea wa masuala ya afya utaongezea mzigo mzito kwa wanaotoa vibali pamoja na kuongezea ukiritimba Zaidi. | | | |
| S7 | Serikali iyalazimishe makampuni ya madini kufanya upembuzi wa masuala ya afya ili kupunguza athari za kiafya. | | | |
| S8 | Leseni za uchimbaji wa madini zisitolewe au kutolewa upya bila ya “kuwa na mpango wa kudhibiti afya ya jamii” | | | |
| S9 | Kujenga uwezo wa kitaalamu ndani ya NEMC wa kufanya upembuzi wa masuala ya afya ya jamii katika pembuzi zote za mazingira. | | | |
| S10 | Kujenga uwezo wa kitaalamu ndani ya Wizara ya Afya wa kufanya upembuzi wa masuala yote ya afya ya jamii. | | | |
| S11 | “Baseline data” za “indicator” za afya lazima zikusanywe ili kufanya upembuzi wa athari za kiafya katika miradi ya madini. | | | |
| S12 | Wizara ya Afya lazima iyapatie makampuni ya madini miongozo inayoeleweka ya namna ya kufanya pembuzi za mambo ya afya. | | | |
| S13 | Watalaamu na wafuatiliaji sharti ajira zao zilindwe kuzuia teuzi za kisiasa na kuzuia taasisi za udhibiti kutoingiliwa kisiasa. | | | |
| S14 | Paundwe kitengo ndani ya Wizara ya Afya cha kufuatilia masuala ya athari za kiafya kwenye miradi mikubwa ya madini. | | | |
| S15 | Makampuni ya madini yakipenda yanaweza kutoa taarifa za athari za kiafya kwa wananchi maeneo ya migodi. | | | |
| S16 | Wizara ya Afya inahitaji fedha zaidi ili kufuatilia utekelezaji wa masuala ya afya ya jamii kwa makampuni ya madini. | | | |
| S17 | Wizara ya Afya lazima iweke wazi kila mwaka matokeo ya ufuatiliaji wa masuala ya afya ya jamii migodini. | | | |
| S18 | Ukaguzi na uchunguzi wa mara kwa mara utakaofanywa na maafisa wa Umma ndiyo utakaoyafanya makampuni ya madini yafuate taratibu za kulinda afya ya jamii. | | | |
| S19 | Serikali ifanyie marekebisho sheria za madini ili kuongezea nguvu ufuatiliaji wa makampuni ya madini yanavyotekeleza masuala ya afya. | | | |
| S20 | Ufuatilia wa athari za afya ya jamii uachiwe makampuni ya madini ambayo yanaweza kutoa taarifa za mwaka za ufuatiliaji. | | | |
| S21 | Uongozi wa Halmashauri za Wilaya na Mikoa ujengewe uwezo wa kufanya uchunguzi mdogo wa masuala ya afya. | | | |
| S22 | Sekta ya afya inahitaji kujihusisha moja kwa moja na ukaguzi wa mara kwa mara wa migodi na kupima uchafuzi wa mazingira na afya. | | | |
| S23 | Serikali inahitajika kutengeneza utaratibu wa kutoa adhabu kwa kutokufuatwa kwa udhibiti na ubora wa afya ya jamii. | | | |
| S24 | Makampuni ya madini lazima yatoe mipango ya ukarabati itakayojumuisha masuala ya afya ya jamii miradi yao inapokoma. | | | |
| S25 | Ianzishwe forum rasmi ya wadau wa serikali, asasi za kiraia, vyuo, na sekta binafsi itakayohakikisha kuna mjadala endelevu wa changamoto za afya katika sekta ya madini. | | | |
| S26 | Ianzishwe “Center of Expertise” katika chuo kikuu au taasisi mojawapo ya utafiti itakachotoa mafunzo kwa watumishi wa umma kufanya upembuzi wa masuala ya afya. | | | |
| S27 | Uratibu kati ya wahusika wa utoaji leseni, na wanaohusika na ufuatiliaji uongezeke Zaidi. | | | |
| S28 | Wizara ya Afya itakuwa na uhusika rasmi katika taratibu za utoaji wa leseni ili kujumuisha masuala ya afya katika upembuzi zilizopo. | | | |
| S29 | Wizara ya Afya i-publish ushauri wake wa kitalaamu kwenye masuala ya upembuzi ili kuepusha kutengwa kwenye mchakato wa maamuzi ya mwisho. | | | |
| S30 | Kuiongezea nguvu tume ya kitaalamu ambapo wote wanaohusika na utoaji wa leseni wanaratibiwa vizuri ili matokeo yao yasije kupuuzwa. | | | |
| S31 | Kampuni za madini sharti zishauriane na watalaamu wa afya, vikundi vya wanawake, AZAKI, na jamii kwa ujumla wanapoanza kufanya upembuzi. | | | |
| S32 | Kampuni za madini zitoe matokeo ya pembuzi zao za masuala ya afya kwa namna rahisi kueleweka na wanachi. | | | |
| S33 | Serikali ina wajibu wa kuwekeza katika huduma za afya ya jamii migodini kwa kutoa vifaatiba, madawa, na mafunzo kwa wafanyakazi wa afya. | | | |
| S34 | Serikali ianzishe uratibu kati ya mifumo ya afya ngazi ya jamii na makampuni ya madini kuhakikisha miradi ya afya ina manufaa. | | | |
| S35 | Kampuni za madini zihamasishwe kutangaza nafasi za miradi ya kijamii ili wahusika katika mfumo wa afya waweze kuwasilisha maombi yao. | | | |
| S36 | Makampuni ya madini yalipie gharama za kufanya tafiti za ufuatiliaji wa mambo ya afya maeneo ya migodini. | | | |
| S37 | Makampuni ya madini yafanye kampeni za mara kwa mara kuelewesha wananchi athari za kiafya pamoja na njia bora za kukuza mazoea bora ya kiafya. | | | |
| S38 | Kampuni za madini zilipie gharama za bidhaa na vifaa (ambulance na vifaa vya maabara kupima afya) katika maeneo ya migodi. | | | |
| S39 | Kampuni za madini ziendeshe kituo cha huduma za afya kwa ajili ya watu wote maeneo ya migodini. | | | |
| S40 | Kampuni za madini sharti zitoe maelezo (ya mambo ya kiafya) kwa umma juu ya malalamiko ya wananchi. | | | |

Source: Authors (The original English version was first published in Proksik et al., 2023)
