# Supplementary material for: Protecting public health in mining: a Q-methodology examination of policy space and reform pathways in Tanzania
Source: BMC Public Health. 2026 Jul 27;26:3036. doi: 10.1186/s12889-026-28398-7 (PMC13688855; doi:10.1186/s12889-026-28398-7)
Supplement: Supplementary file 2 [file 12889_2026_28398_MOESM2_ESM.docx]

## Table A.2: Factor characteristics

|  | Factor 1 | Factor 2 | Factor 3 |
| --- | --- | --- | --- |
| Number of defining variables | 8.00 | 7.00 | 3.00 |
| Average relative coefficient | 0.80 | 0.80 | 0.80 |
| Composite reliability | 0.97 | 0.966 | 0.923 |
| Standard error of factor *z* scores | 0.173 | 0.184 | 0.277 |

Source: Authors
