# Supplementary material for: Protecting public health in mining: a Q-methodology examination of policy space and reform pathways in Tanzania
Source: BMC Public Health. 2026 Jul 27;26:3036. doi: 10.1186/s12889-026-28398-7 (PMC13688855; doi:10.1186/s12889-026-28398-7)
Supplement: Supplementary file 3 [file 12889_2026_28398_MOESM3_ESM.docx]

## Table A.3: List of study participants

| TZ01 | Senior official, Ministry of Health (MoH) |
| --- | --- |
| TZ02 | Zonal manager, National Environmental Council (NEMC) |
| TZ03 | Senior executive, private consultancy firm I |
| TZ04 | Senior official, Ministry of Health (MoH) |
| TZ07 | Senior public health official, President’s Office, Regional Administration and Local Government (PO-RALG) |
| TZ08 | Senior official, Ministry of Mining (MoM) |
| TZ09 | Senior official, Ministry of Mining (MoM) |
| TZ10 | Technical expert, Ministry of Mining (MoM) |
| TZ11 | District-level public health official, PO-RALG |
| TZ12 | District-level environmental management official, PO-RALG |
| TZ15 | Senior executive, national civil society organization |
| TZ16 | Senior public health official, PO-RALG |
| TZ17 | District-level environmental management official, PO-RALG |
| TZ18 | Zonal manager, National Environmental Council (NEMC) |
| TZ19 | District-level public health official, PO-RALG |
| TZ21 | Local academia member, University of Dar es Salaam (UDSM) |
| TZ23 | Local representative, international development NGO |
| TZ24 | Senior official, National Environmental Council (NEMC) |
| TZ25 | Senior official, National Environmental Council (NEMC) |
| TZ26 | High-ranking official, Ministry of Mining (MoM) |
| TZ28 | Senior representative, Tanzania Chamber of Mines |
| TZ29 | Representative, large-scale mining company |
| TZ30 | Environmental management professional, private consultancy firm II |

Source: Authors
