# Supplementary material for: Protecting public health in mining: a Q-methodology examination of policy space and reform pathways in Tanzania
Source: BMC Public Health. 2026 Jul 27;26:3036. doi: 10.1186/s12889-026-28398-7 (PMC13688855; doi:10.1186/s12889-026-28398-7)
Supplement: Supplementary file 4 [file 12889_2026_28398_MOESM5_ESM.docx]

**Protecting Public Health in Mining**

A Q-Methodology Examination of Policy Space and Reform Pathways in Tanzania

**Supplementary File: Q-Methodology Reporting Checklist (Dieteren et al., 2023)**

| Item | Requirement | Evidence | Location |
| --- | --- | --- | --- |
| 1.1 | Concourse source | Literature, regulation review, expert interviews, focus groups | pp.10-11 |
| 1.2 | Item sampling | 234 statements reduced to 40 | p.11 |
| 1.3 | Type of items | Policy statements | pp.10-11 |
| 1.4 | Number of items | 40 statements | p.11 |
| 1.5 | Pilot | Pilot with stakeholder representatives, results described | p.11 |
| 2.1 | Participant selection | Purposeful sampling, motivated | p.12 |
| 2.2 | Number participants | 23 participants | p.12 |
| 2.3 | Mode interview | Face-to-face | p.13 |
| 2.4 | Type interview | Individual administration | p.13 |
| 2.5 | Ranking instruction | Pile sort then +5 to -5 ranking | p.13 |
| 2.6 | Sorting grid | Forced distribution grid, provided as a figure | Fig.2 |
| 3.1 | Factor analysis | 3-factor extraction, varimax rotation | p.13 |
| 3.2 | Factor selection | Kaiser-Guttman, scree, variance tests | p.13 |
| 3.3 | Bipolar factors | F3 split into F3a/F3b | p.14 |
| 3.4 | Interpretation | Interview data and expert verification | p.15 |
| 3.5 | Software | KADE v1.2.1 | p.13 |
| 4.1 | Factor solution | Solution statistics reported incl. appendix | p.14+ |
| 4.2 | Factor arrays | Tables and appendix | Results |
| 4.3 | Distinguishing/consensus | Supplementary files | Appendix |
| 4.4 | Factor descriptions | Narrative factor descriptions | Results |
